# A Study of the Interaction of a New Benzimidazole Schiff Base with Synthetic and Simulated Membrane Models of Bacterial and Mammalian Membranes

**DOI:** 10.3390/membranes11060449

**Published:** 2021-06-16

**Authors:** Alberto Aragón-Muriel, Yamil Liscano, David Morales-Morales, Dorian Polo-Cerón, Jose Oñate-Garzón

**Affiliations:** 1 Laboratorio de Investigación en Catálisis y Procesos (LICAP), Departamento de Química, Facultad de Ciencias Naturales y Exactas, Universidad del Valle, Cali 760031, Colombia; alberto.aragon@correounivalle.edu.co; 2 Grupo de Investigación en Química y Biotecnología (QUIBIO), Facultad de Ciencias Básicas, Universidad Santiago de Cali, Cali 760035, Colombia; yamil.liscano00@usc.edu.co; 3 Instituto de Química, Universidad Nacional Autónoma de México, Cd. Universitaria, Circuito Exterior, Coyoacán, Mexico D.F. 04510, Mexico; damor@unam.mx

**Keywords:** model membranes, molecular dynamics, calorimetry, Schiff base, imine, benzimidazole, 2,4-dihydroxybenzaldehyde

## Abstract

Biological membranes are complex dynamic systems composed of a great variety of carbohydrates, lipids, and proteins, which together play a pivotal role in the protection of organisms and through which the interchange of different substances is regulated in the cell. Given the complexity of membranes, models mimicking them provide a convenient way to study and better understand their mechanisms of action and their interactions with biologically active compounds. Thus, in the present study, a new Schiff base (*Bz-Im*) derivative from 2-(*m*-aminophenyl)benzimidazole and 2,4-dihydroxybenzaldehyde was synthesized and characterized by spectroscopic and spectrometric techniques. Interaction studies of (*Bz-Im*) with two synthetic membrane models prepared with 1,2-dimyristoyl-sn-glycero-3-phosphocholine (DMPC) and DMPC/1,2-dimyristoyl-sn-glycero-3-phosphoglycerol (DMPG) 3:1 mixture, imitating eukaryotic and prokaryotic membranes, respectively, were performed by applying differential scanning calorimetry (DSC). Molecular dynamics simulations were also developed to better understand their interactions. In vitro and in silico assays provided approaches to understand the effect of *Bz-Im* on these lipid systems. The DSC results showed that, at low compound concentrations, the effects were similar in both membrane models. By increasing the concentration of *Bz-Im*, the DMPC/DMPG membrane exhibited greater fluidity as a result of the interaction with *Bz-Im*. On the other hand, molecular dynamics studies carried out on the erythrocyte membrane model using the phospholipids POPE (1-palmitoyl-2-oleoyl-sn-glycero-3-phosphoethanolamine), SM (N-(15Z-tetracosenoyl)-sphing-4-enine-1-phosphocholine), and POPC (1-palmitoyl-2-oleoyl-sn-glycero-3-phosphocholine) revealed that after 30 ns of interaction, both hydrophobic interactions and hydrogen bonds were responsible for the affinity of *Bz-Im* for PE and SM. The interactions of the imine with POPG (1-Palmitoyl-2-Oleoyl-sn-Glycero-3-Phosphoglycerol) in the *E. coli* membrane model were mainly based on hydrophobic interactions.

## 1. Introduction

Biological membranes are essential for life since they regulate the entry and exit of nutrients, neurotransmitters, and drugs [1]. Biological membranes contain three main types of lipids: phospholipids, glycolipids, and cholesterol. Phospholipids are, in turn, divided into different groups according to the structural properties of the polar head: phosphatidylcholine (PC), sphingomyeline (SM), and phosphatidylethanolamine (PE) are common lipids present in eukaryotic cell membranes [2].

Since drugs operate through different mechanisms when their main targets are intracellular and, therefore, they must penetrate the cell membrane to exert their pharmacological action, it is essential to understand drug–membrane interactions [3]. However, the complexity of the structure and functionality of cell membranes, as well as the highly dynamic nature of lipid–lipid and lipid–protein interactions, make drug–membrane system studies difficult [4].

Thus, artificial model membrane systems were developed to facilitate the understanding of the effects of membrane lipids on drug transport and absorption in cells, drug activity, and even drug toxicity [5,6]. Within the different types of model membranes, liposomes are highly suitable for permeability research and drug delivery systems. Additionally, they allow for the use of various thermoanalytical and spectroscopic techniques—such as isothermal titration calorimetry (ITC), differential scanning calorimetry (DSC), Fourier transform infrared spectroscopy (FT-IR), fluorescence spectroscopy, and nuclear magnetic resonance (NMR) methods—to study biophysical interactions of the drug–membrane complex [7,8,9,10,11].

In studies that make use of model membranes, saturated or unsaturated PC species are used to mimic eukaryotic cells, while the PC/PG model is used to mimic bacterial membranes [12]. Studies have revealed that the effect of azole compounds on model membranes [13,14], including membranes based on the PC/PG species, is controlled by drug–membrane interactions which depend on the length, unsaturation, and head group of the phospholipids, as well as the surface charge of the target cell [15]. In particular, compounds derived from benzimidazole interact with model membranes of human erythrocytes using the passive diffusion method [16]. In silico studies demonstrated that hydroxyl groups present in derivatives of benzimidazole decrease the hydrophobic character of DPPC (dipalmitoylphosphatidylcholine) model membranes and interact with the phosphate group of the polar heads present in the membrane [17].

Studies including compounds with the benzimidazole motif on their structures are interesting for the scientific community, not only because of their known antibacterial and cytotoxic properties [18,19,20], but because of the high conjugation that they exhibit when forming Schiff bases, improving their electronic characteristics and often conferring fluorescent properties that facilitate the monitoring of morphology in microorganisms subjected to these types of drugs [21].

Hence, based on the antibacterial and cytotoxic properties that Schiff bases obtained from (1*H*-benzimidazol-2-yl)anilines have demonstrated [22,23,24], this article describes the synthesis and characterization of 4-(((3-(1*H*-benzo[*d*]imidazol-2-yl)phenyl)imino)methyl)benzene-1,3-diol and the study of its possible mechanism of interaction with bacterial and mammalian membrane models by analyzing the thermodynamic profiles of the phase transition by DSC. In addition, in order to explore the *Bz-Im* effect on the thermotropic behavior of bacterial and mammalian systems, proper membrane models consistent with experimental membrane models were developed. Thus, the interaction of the imine towards model membranes of human erythrocytes and *E. coli* were described from results by molecular dynamics (MD) simulations.

## 2. Materials and Methods

### 2.1. Synthesis of Benzimidazole Schiff Base

#### 2.1.1. Materials

All chemical reagents used for the synthesis of benzimidazole and subsequent imine were used as received and without further purification. Elemental analyses were performed using Flash EA 1112 Series CHN Analyzer. A Shimadzu Affinity 1 FT-IR spectrometer was used to obtain the infrared spectra. IR data are reported using the following abbreviations: vs = very strong; s = strong; m = medium; w = weak; sh = shoulder; br = broad. ^1^H and ^13^C{^1^H} NMR spectra were obtained on a Bruker Avance II 400 spectrometer using DMSO-*d*_6_ as a solvent at 25 °C. The following abbreviations were used: s = singlet; d = doublet; t = triplet; m = multiplet. The mass spectrum of the benzimidazole was recorded on a Shimadzu-GCMS-QP2010 at 70 eV by electronic impact (EI) ionization, while the mass spectrum of the derived imine was obtained by direct analysis in real time (DART) ionization system on a JEOL AccuTOF JMS-T100LC.

#### 2.1.2. Synthesis of 2-(m-aminophenyl)benzimidazole (*Bz*)

The condensation reaction of *o*-phenylenediamine with *m*-aminobenzoic acid was carried out following a similar methodology to that previously reported [25]. *o*-phenylenediamine (0.54 g, 5 mmol), *m*-aminobenzoic acid (0.69 g, 5 mmol), and polyphosphoric acid were mixed and stirred for 2.5 h at 180 °C. After this time, the resulting reaction mixture was allowed to cool and then neutralized with sodium carbonate (20%) before the resulting solution was filtered. The violet precipitate was then washed with distilled water, purified with activated carbon, and recrystallized in ethanol to give the final product as a beige powder. Yield: 0.87 g, 83%. C_13_H_11_N_3_ (209.25 g·mol^−1^): Calc. C, 74.62; H, 5.30; N, 20.08. Found: C, 74.58; H, 5.36; N, 20.09%. IR (ATR cm^−1^): 3826 w, 3739 w, 3425 w, 3321 w, 3205 w, 2328 w, 1683 w, 1616 vs. 1566 s, 1510 s, 1463 s, 1394 m, 1346 m, 1234 m, 1062 m, 945 w, 893 m, 835 w, 750 vs. ^1^H NMR (DMSO-*d*_6_) δ (ppm) 12.70 (s, 1H), 7.62 (d, *J* = 7.0 Hz, 1H), 7.49 (d, *J* = 7.0 Hz, 1H), 7.43 (s, 1H), 7.28 (d, *J* = 7.5 Hz, 1H), 7.23–7.11 (m, 3H), 6.68 (d, *J* = 7.8 Hz, 1H), 5.30 (s, 2H). MS (EI, *m*/*z*): 209.

#### 2.1.3. Synthesis of 4-(((3-(1*H*-benzo[*d*]imidazol-2-yl)phenyl)imino)methyl)benzene-1,3-diol (*Bz-Im*)

As was the case for the intermediate *Bz*, the imine *Bz-Im* was synthesized based on previously reported procedures [26,27]. 2-(*m*-aminophenyl)benzimidazole (0.52 g, 2.5 mmol), 2,4-dihydroxybenzaldehyde (0.35 g, 2.5 mmol), and methanol were mixed and set to reflux under stirring for 2 h. After this time, the yellow precipitate obtained was washed with cold water and then dried under vacuum for 4 h. Yield: 0.72 g, 87%. C_20_H_15_N_3_O_2_ (329.36 g·mol^−1^): Calc. C, 72.94; H, 4.59; N, 12.76. Found: C, 72.83; H, 4.56; N, 12.91%. IR (ATR cm^−1^): 3381 w, 3046 w, 1891 w, 1600 s, 1567 sh, 1512 w, 1494 vs. 1451 m, 1384 s, 1268 sh, 1259 s, 1222 m, 1143 s, 1114 m, 963 w, 910 m, 856 s, 807 vs. 725 vs. ^1^H NMR (DMSO-*d*_6_) δ (ppm) 13.46 (s, 1H), 12.98 (s, 1H), 10.34 (s, 1H), 8.94 (s, 1H), 8.22–8.01 (m, 2H), 7.70–7.45 (m, 5H), 7.24 (s, 2H), 6.45 (dd, *J* = 8.4, 2.3 Hz, 1H), 6.35 (d, *J* = 2.3 Hz, 1H). ^13^C{^1^H} NMR (DMSO-*d*_6_) δ (ppm) 163.7, 163.5, 163.2, 151.3, 149.3, 144.2, 135.5, 135.1, 131.83, 130.6, 124.6, 123.2, 122.8, 122.3, 119.6, 119.4, 112.6, 111.9, 108.5, 102.9. MS (DART+) *m*/*z*: 330.

### 2.2. Interaction with Models of Synthetic Membranes

#### 2.2.1. Membrane Preparation

Model membranes mimicking mammalian and bacterial membranes were prepared following the methodology reported previously [28]. Thus, DMPC and DMPG lipids at a molar ratio of 3:1 were dissolved in chloroform/methanol (2:1 *v*/*v*) to imitate gram-negative bacterial membranes [2], while the DMPC lipid alone was dissolved in chloroform/methanol (2:1 *v*/*v*) to imitate zwitterionic human cell membranes. The lipid mixture was first dried under a stream of nitrogen and then under vacuum for a further three hours. The hydration process was performed by preparing different concentrations of *Bz-Im* using HEPES buffer (25 mM HEPES, pH 7.0; 100 mM NaCl and 0.2 mM EDTA), which were added to the existing dry lipid mixture and vigorously shaken with a vortex for 2 min before incubation for 10 min at 37 °C above the phase transition temperature (T_m_). The multilamellar vesicles (MLVs) were obtained after repeating the shaking and incubation process three times [29].

#### 2.2.2. Differential Scanning Calorimetry

For the acquisition of thermograms by DSC analysis, a TA instrument DSC Q25 was used. Multillamellar vesicles (MLVs) were prepared using 2 mg of lipids hydrated with *Bz-Im* diluted in HEPES buffer to give three *Bz-Im*–lipid ratios: 1:50, 1:25, and 1:10. HEPES buffer was used as a reference solution. The samples were placed and subsequently sealed within standard aluminum DSC pans, and were analyzed over a range of 10 to 35 °C at a heating rate of 1 °C/min. Trios software (TA Instruments) was used to obtain the phase transition temperature (T_m_), the transition enthalpy (ΔH), and the full width at half-maximum from thermograms (FWHM, ΔT_m_/2).

### 2.3. Molecular Dynamics (MD) Studies

#### 2.3.1. Construction of the 3D Structure of *Bz-Im*

The 3D structure of *Bz-Im* was drawn using Chemdraw software (https://chemdrawdirect.perkinelmer.cloud/js/sample/index.html, access date: 8 March 2021). The *Bz-Im* structure was optimized using the universal force field (UFF) [30] and the steepest descent algorithm with Avogadro version 1.2 software. 

#### 2.3.2. Erythrocyte Membrane Construction

The zwitterionic model membrane of erythrocyte cells, which was mimicked by zwitterionic phosphatidylcholine for DSC assays, was built with the CHARMM-GUI [31] platform using, as a basis, the phospholipid composition mentioned by Texeira et al. [32]. The phospholipid proportions used were 20 units of POPE, 40 units of SM, and 40 units of POPC, which were distributed both in the upper and lower layer of the membrane. For the placement of the ions, the Monte Carlo method was used with a concentration of 0.15 M NaCl. In addition, a water thickness of 22.5 Å, and a force field for the entire CHARMM36m system was used [33]. The files were prepared to minimize energy, balance, and dynamics with GROMACS [34] at 310 K.

#### 2.3.3. Construction of Gram-Negative Bacterial Membrane Models

The membrane model systems for *E. coli* were constructed based on the data reported by Epand et al. [35], using the same distribution published by Liscano et al. [36] for a gram-negative membrane model system (POPE = 80 units and POPG = 20 units), with CHARMM-GUI software [31].

#### 2.3.4. Implementing Molecular Dynamics

The minimization energy of the erythrocyte model membrane and *E. coli* membrane system with the ligand *Bz-Im* was adjusted with the steepest descent algorithm in 5000 steps using the Verlet cutoff scheme. Equilibration was performed for 2 ns using the Berendsen algorithm to equilibrate the temperature and pressure of the system. Molecular dynamics were run for 10 ns at 310 K using the Nose–Hoover and Parrinello–Rahman algorithms to adjust temperature and pressure.

Gromacs software version 2020.1 [34] was used for the molecular dynamics simulation of gram-negative bacterial and erythrocyte membrane models. The CHARMM36m force field [33] was used for the simulation. For the localization of the ions, the Monte Carlo method was used with 0.15 M NaCl in water 22.5 Å thick. For the energy minimization, the steepest descent algorithm was used, running for 1 ns. Using the Berendsen algorithm, the system was adjusted to a temperature of 310 K with an equilibration of 2 fs/step for 300 ps to 155,000 n-steps. Once the system was equilibrated the molecular dynamics were run for 30 ns for both erythrocyte and *E. coli* model membranes, using the Nose–Hoover and Parrinello–Rahman algorithms to adjust the temperature and pressure.

#### 2.3.5. Interaction Analysis

Gromacs was used to obtain the hydrogen bonds between *Bz-Im* and the phospholipids of each membrane model system within 30 ns. PyMOL PDB files were obtained for each membrane system at six different times: 1, 5, 10, 20, 25, and 30 ns. These files were used to visualize and analyze the interactions between the different components of each model system and the *Bz-Im* using Discovery Studio Visualizer software. 

## 3. Results and Discussion

### 3.1. Schiff Base (Imine) Characterization

**Scheme 1:**
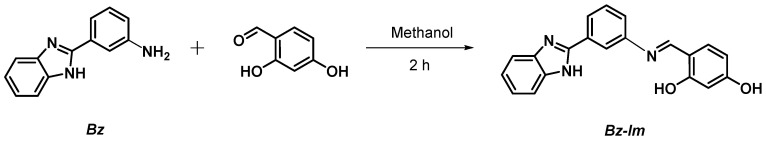
Synthesis of *Bz-Im*.

The synthesis of Schiff base 4-(((3-(1*H*-benzo[*d*]imidazol-2-yl)phenyl)imino)methyl)benzene-1,3-diol **(*Bz-Im*)** was carried out from the reaction between 2,4-dihydroxybenzaldehyde and 2-(*m*-aminophenyl)benzimidazole **(*Bz*)** (Scheme 1).

Compound *Bz-Im* was obtained in high yield (87%) as a yellow powder, and its structure was unequivocally determined by mass spectrometry, elemental analyses (C, H, and N), infrared spectroscopy, and nuclear magnetic resonance spectroscopy. The FT-IR spectra of *Bz-Im* showed bands corresponding to υ (3425 and 3321 cm^−1^) stretching and deformation δ (1616 cm^−1^) vibrations of the amino group in *Bz* disappeared after the formation of the imine, while a new band was observed at 1600 cm^−1^, characteristic for this type of compound, confirming the formation of the N=CH bond [37]. In the infrared spectrum, the characteristic band of the stretching vibration υ_C-O_ at 1259 cm^−1^ was also observed (phenolic fragment). In addition, the ^1^H NMR spectrum of *Bz-Im* showed the characteristic signal of the imine group at 8.94 ppm, while signals due to the aromatic protons were observed around 8.25–6.25 ppm [22,27]. Additionally, the ^13^C{^1^H} NMR spectra exhibited a typical signal due to the imine carbon at ~163 ppm. This one-dimensional NMR analysis was further supported with two-dimensional studies that can be consulted in Figures S4, S6, and S7 of the supplementary material. Finally, analysis by mass spectrometry (DART+) afforded a spectrum exhibiting the peak due to the molecular ion [M+1] at 330 *m*/*z* (Figure S9). Elemental analysis results were also in agreement with the proposed structure.

### 3.2. Model Membrane Studies

#### 3.2.1. Thermotropic Behavior of Synthetic Model Membranes

**Figure 1:**
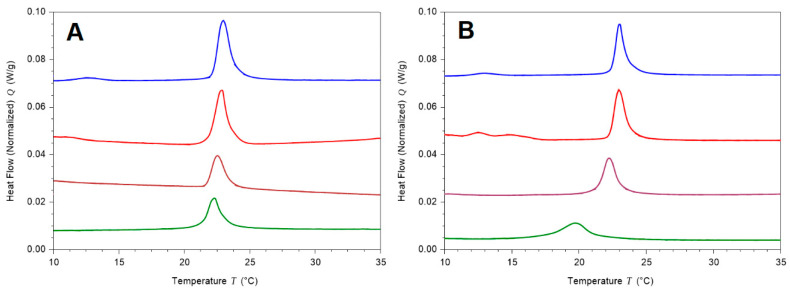
Thermotropic profile of MLVs made up of (**A**) DMPC and (**B**) DMPC-DMPG (3:1), under different amounts of imine. Compound–lipid molar ratios: 0:1 (---); 1:50 (---); 1:25 (---); and 1:10 (---).

The membrane models included mammalian-like membranes consisting of the phospholipid DMPC and bacterial-like membranes consisting of a 3:1 ratio of DMPC:DMPG. By gradually heating the vesicles without compound, the acquisition of thermotropic profiles was achieved (Figure 1), where a pre-transition endothermic peak was observed at 12.94 °C for the DMPC systems and at 12.71 °C for the DMPC: DMPG 3:1 mixture. PCs have a fairly bulky headgroup, creating a size mismatch with their acyl chains, especially below the main phase transition [38]. As the temperature increases, the main transition peak emerges at 23.02 °C for the two systems mentioned above—these results being consistent with those reported previously [28].

By addition of *Bz-Im* in a 1:50 compound–lipid molar ratio, changes were observed in the pre-transition of both systems (Table 1), suggesting that this compound affects the transition from a flat membrane phase (Lβ) to a ripple phase (Pβ) as a result of the changes in size mismatch with the phosphatydilcholine headgroup hydration [38]. Interestingly, in the pre-transition from the DMPC: DMPG mixture, two subtle peaks emerged (Figure 1B). Riske et al. [38] described that up to 20% fluid lipid population were detected between the pre- and the main transitions. In addition, gauche conformers are introduced into the acyl lipid chains in the pre-transition [39]. By increasing the concentration of *Bz-Im* (1:25 compound–lipid molar ratio), the pre-transition is abolished, the T_m_ is changed moderately, and the size of the peak of the main transition decreases as the width of the peak increases in both lipid systems (Figure 1). The surface of the bilayer must be considered as a set of several phospholipid “clusters”, where all the molecules of each cluster exhibit a simultaneous behavior in the transition. This cooperative property of the melting process defines more sharp and symmetrical curves at the transition peak. In this way, *Bz-Im* is able to penetrate into the bilayer since the “clusters” noticeably increase in number [40]. ΔT_m_/2 is a relative measure of molecular cooperativity and it linearly increases with the concentration of *Bz-Im*, ranging from 0.98 to 1.14 °C and from 0.73 to 1.08 °C for DMPC and DMPC:DMPG systems, respectively, suggesting the insertion of “free volumes” into the bilayer structure [40]. Therefore, the full width at half-maximum of the main transition peak is a variable that indicates how cooperative the phospholipids are when they undergo a transition [41]. Similarly, the enthalpy of transition is considerably and moderately reduced in DMPC and DMPC/DMPG, respectively (Table 1), suggesting that the addition of anionic lipids to the zwitterionic lipids avoids greater alterations to the interactions between lipid acyl chains, i.e., the disruption of *trans-gauche* isomerization and the inter- and intramolecular van der Waals interactions [7]. This can be explained by a strong adhesion of the *Bz-Im* on the anionic surface by phosphatidylglycerol at this concentration, where the small size and the reduced flexibility of *Bz-Im* prevents their hydrophobic moieties from being inserted inside the bilayer.

At the maximum concentration of *Bz-Im*, the size of the peaks representing the main transition were decreased. Interestingly, a pronounced change in T_m_ and in the width of the peak was observed only in the DMPC/DMPG mixture (Figure 1), suggesting that *Bz-Im* at this concentration increases the fluidity and the lateral phase separation in membranes that mimic those of bacteria. In fact, fluidity is known to increase in response to an increase in the lateral diffusion rates of lipid molecules [42], and has been related to alterations within the hydrophobic nucleus of the bilayer [43]. Thus, it is likely that more moles of *Bz-Im* bind to a smaller unit area in DMPC/DMPG than to an entire surface area of DMPC, reaching a lower threshold concentration due to the presence of DMPG, which is related to the degree of insertion of compounds inside the bilayer [44].

#### 3.2.2. Analysis of Molecular Dynamics

**Figure 2:**
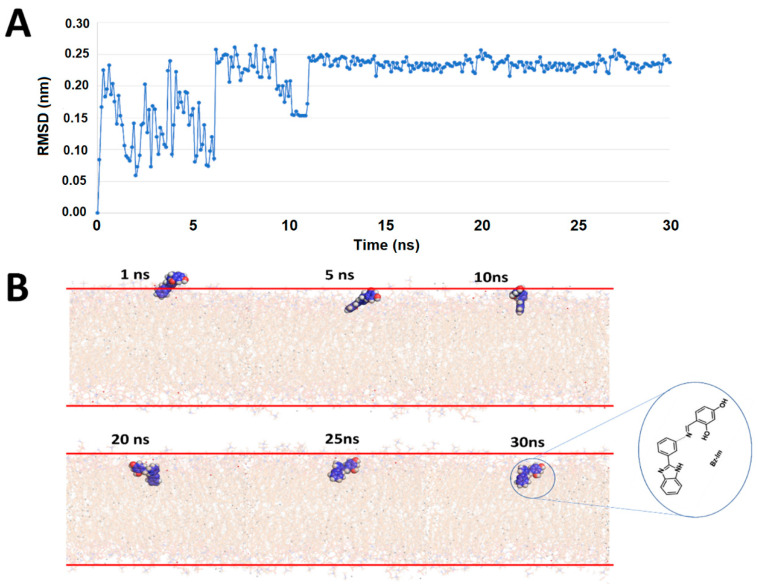
Behavior of *Bz-Im* in the erythrocyte membrane model system for 30 ns. (**A**) Root mean square deviation (RMSD) of *Bz-Im* in the erythrocyte membrane system at 30 ns. (**B**) *Bz-Im* movement from the surface of the erythrocyte membrane during the 30 ns.

In order to understand the results of the thermotropic profile of the membrane models, the interaction between *Bz-Im* and the molecular models of erythrocyte zwitterionic membranes was analyzed by molecular dynamics. Figure 2 shows the root mean square deviation (RMSD) of *Bz-Im* in the erythrocyte membrane over 30 ns. From 0 to 10 ns a continuous variation of the RMSD is observed (Figure 2A), suggesting the whole structure fluctuates, or it might reflect only large displacements of a small structural subset within an overall rigid structure [45] as a result of the loss of bonds between phospholipids and the formation of *Bz-Im*-membrane interactions during the insertion and adjustment of the compound inside the polar head of the phospholipids in these first nanoseconds (Figure 2B). A stabilization of the structural configuration of *Bz-Im* is observed from 10 ns that coincides with the penetration of *Bz-Im* inside the polar region of the membrane, indicating that it does not bring any considerable changes to the overall conformation of the system over 30 ns MD trajectories.

**Figure 3:**
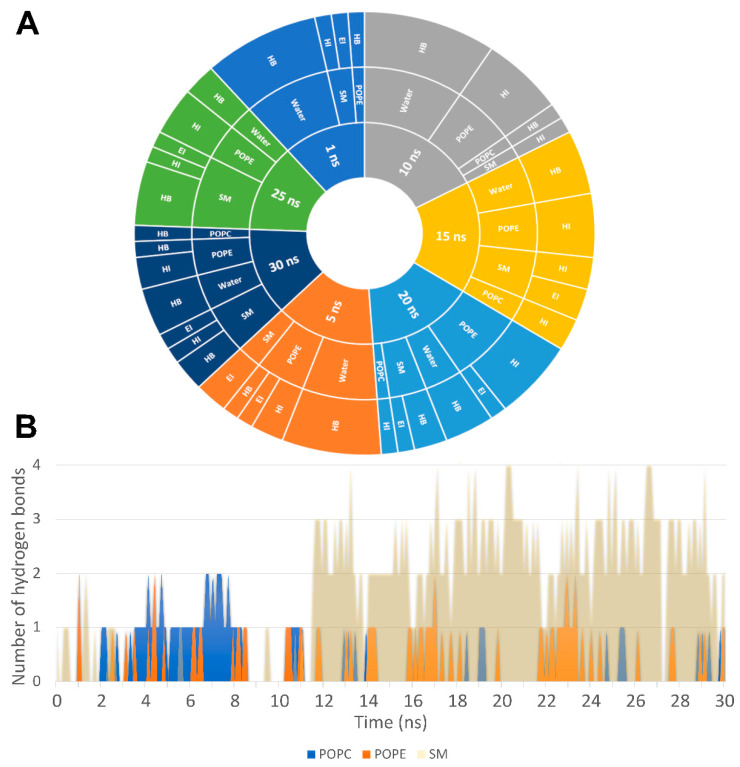
Intermolecular interactions between *Bz-Im* and the erythrocyte membrane system (**A**) at 1 ns (blue color), 5 ns (orange color), 10 ns (gray color), 15 ns (yellow color), 20 ns (light blue color), 25 ns (green color), and 30 ns (dark blue color). HB, hydrogen bond; HI, hydrophobic interaction; E, electrostatic interactions (**B**) Hydrogen bonds between *Bz-Im* and the phospholipids of the system. SM, sphingomyelin; POPC, phosphatidylcholine; POPE, phosphatidylethanolamine.

Figure 3 shows the interactions of *Bz-Im* with the components of the erythrocyte membrane model system, observing a greater number of hydrogen bond-type interactions with water molecules in the first nanoseconds after starting the simulation due to the interaction with the membrane-water interfacial region. These interactions with water decrease as time progresses from 10 to 30 ns. Conversely, interactions with SM and POPE increase as time progresses since they are the phospholipids that most interact with *Bz-Im* in comparison with POPC (Figure 3) due to the presence of the amine group in POPE which forms the additional bonds [46]. In addition, PC and SM have the same polar head but differ in their interfacial structures due to a decrease in headgroup size of the SM causing closer molecular packing. The increased interactions at the membrane interface could influence increased affinity of *Bz-Im* for SM as compared with POPC [47]. Both hydrophobic interactions and hydrogen bonds are responsible for the affinity of *Bz-Im* for PE and SM (Figure 3A), suggesting that the C-N bonds are oriented towards the core of the bilayer and the O-H groups are oriented towards the water phase (Figure 2B).

Figure 3B shows the number of hydrogen bonds between *Bz-Im* and phospholipids through time. Interestingly, interactions with SM emerge from 11 nanoseconds, remaining up to 30 ns, suggesting an initial selectivity for PE, which would directly interact with the NH_3_^+^ moiety and not with the quaternary amine group of choline. When replacing PE by PC, this could arise from the sole removal of the hydrogen-bonding capability of the headgroup [48]. However, there are also hydrogen bonds between *Bz-Im* and POPC from 2 to 8 ns while it was entering the membrane. This could be due to the larger size of the PC polar head, compared with those of PE and SM, occupying a greater volume and exhibiting a better probability of initial contact with *Bz-Im*. Once it is internalized, a strong interaction with sphingomyelin is maintained. It was revealed that the OH-group or NH-function of SM play an important role in hydrogen bonding interactions with foreign compounds [49].

Based on the above, *Bz-Im* can be buried up to the interfacial region of the outer monolayer of a zwitterionic membrane, decreasing the van der Waals interactions between the phospholipids, while interactions that require less heat to undergo the transition are formed (Figure 1A). Hence, it is likely that *Bz-Im* exhibits cytotoxic activity against mammalian cells, a property that could be explored and exploited for tumor cells (Supplementary materials, Section 2).

**Figure 4:**
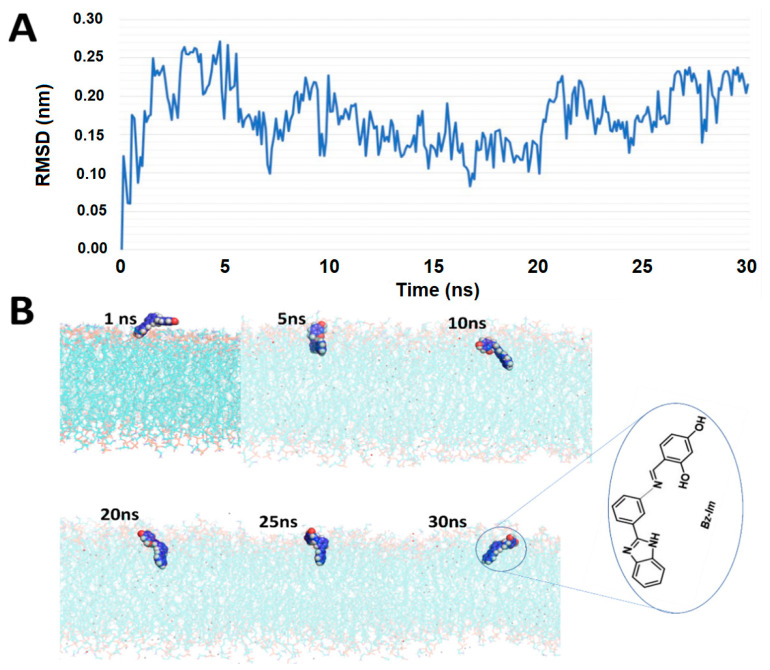
Behavior of *Bz-Im* in the *E. coli* membrane model system for 30 ns. (**A**) Root mean square deviation (RMSD) of *Bz-Im* in the *E. coli* membrane model system for 30 ns. (**B**) *Bz-Im* movement from the surface of the *E. coli* membrane model system during the 30 ns.

Figure 4 shows the behavior of *Bz-Im* on the *E. coli* model membrane for 30 ns. The RMSD reveals that during the first 6 ns there is a great structural variation of *Bz-Im* related to its location between the surface of the membrane and the water phase, which causes intermittent contact with both water molecules and POPE or POPG. Between 10 and 20 ns there is a slight structural variation of *Bz-Im* and during this time the molecule remains submerged in the membrane. Again, *Bz-Im* re-emerges on the membrane surface between the aqueous and lipid phase, which is reflected in a greater structural deviation between 20 and 21 ns as a result of different solvation changes. Finally, *Bz-Im* is internalized again inside the head group of phospholipids between 28 and 30 ns. Unlike the erythrocyte membrane, the position of the *Bz-Im* in the *E. coli* membrane model fluctuated highly during the 30 ns. To explain this, it must be considered that the erythrocyte membrane is made up of only 20% POPE while that of *E. coli* has 80% POPE. The NH_3_^+^ group of PE binds with oxygen from unesterified phosphate by very close contacts [50]. Subsequently, the bonds between adjacent phosphates form a very compact network of PE polar heads in the surface of the membrane, hindering the access of *Bz-Im* and reorienting it within the lipid phase over the first 20 ns. On the other hand, the glycerol moiety of PG mimics the solvation water of the phosphate group [51]. This internal hydrogen bonding makes the hydrogen bonding between the foreign compounds and the phosphate less favorable than when the phosphate is linked to cholines. Thus, phosphate must be shielded by the glycerol moiety in PG, avoiding the formation of hydrogen bonds with compounds at the expense of dehydration [52].

**Figure 5:**
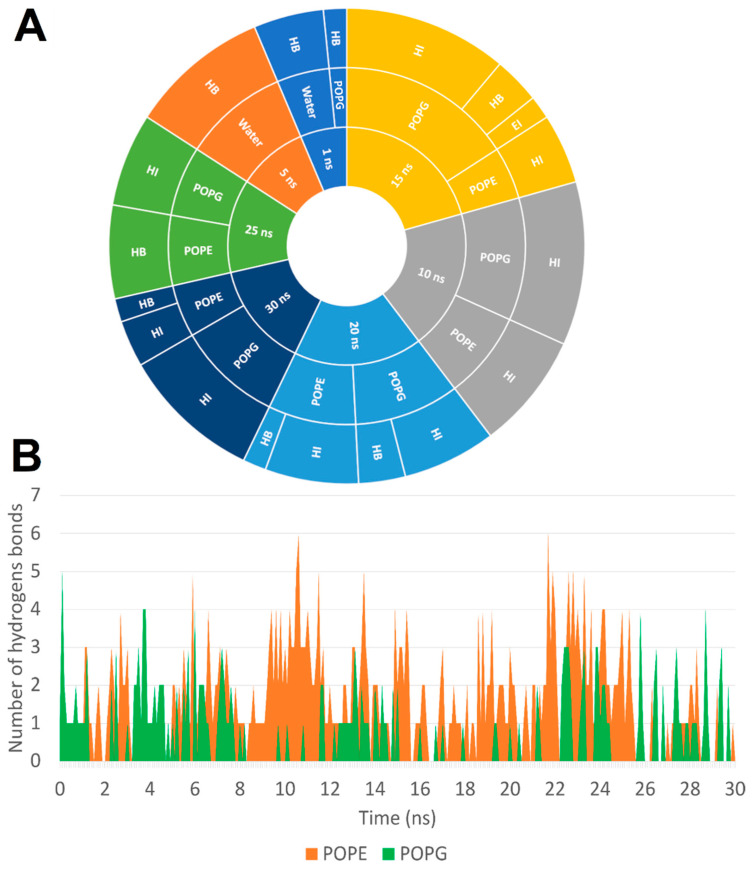
Intermolecular interactions between *Bz-Im* and the *E. coli* membrane model (**A**) at 1 ns (blue color), 5 ns (orange color), 10 ns (gray color), 15 ns (yellow color), 20 ns (light blue color), 25 ns (green color), and 30 ns (dark blue color). HB, hydrogen bond; HI, hydrophobic interaction; EI, electrostatic interactions. (**B**) Hydrogen bonds between *Bz-Im* and the phospholipids of the system. POPE, phosphatidylethanolamine; POPG, phosphatidylglycerol.

Figure 5A shows a greater interaction with water molecules through hydrogen bonds in the first 15 nanoseconds. From 10 ns there is a tendency to form hydrophobic bonds with both phospholipids; this type of interaction is maintained until 30 ns, suggesting that *Bz-Im* must penetrate at least partially into the hydrophobic core of the phospholipid bilayer. As the system is highly dynamic, it is probable that the hydrophobic moiety of the aromatic heterocyclic ring interacts with the acyl chains in a region close to the interface (Figure 4B). The partial insertion into the hydrophobic core would be responsible for the increase in the fluidity described in bacterial model membranes at a 1:10 *Bz-Im*:lipid molar ratio (Figure 1B), since interaction of compounds with the phospholipid acyl chains has been related to a net fluidizing effect of the apolar part of the bilayer [43]. Finally, Figure 5B shows the number of hydrogen bonds formed between *Bz-Im* and the phospholipids of the *E. coli* model membrane; a similar interaction with POPE and POPG was observed, except for at 10 and 22 ns when interaction peaks with POPE of up to six hydrogen bonds were found. The amine group of PE which forms additional bonds [46] would be favorably oriented towards the polar groups of *Bz-Im* at both times. 

## 4. Conclusions

A benzimidazole-derived imine was successfully synthesized and characterized by spectroscopic and spectrometric techniques. The thermotropic profiles indicate that this compound can bind both to DMPC and DMPC:DMPG (3:1), which mimic the mammalian and bacterial membranes, respectively. Our results suggest that *Bz-Im* can increase the fluidity in membranes that mimic those of bacteria, which might be correlated with their potential antibacterial activity, representing a valuable contribution towards the further design of antimicrobial compounds based on benzimidazole-derived imine analogues. Preliminary evidence shows that compound *Bz-Im* has affinity for PC phospholipids, suggesting that this molecule may have effects against normal human cells, that is, the *Bz-Im* compound could be cytotoxic toward these cells. At 30 ns of simulation, hydrogen bonding interactions between *Bz-Im* and SM prevail in erythrocyte membrane models, while in *E. coli* membrane models the hydrophobic interactions between *Bz-Im* and PG/PE play an important role on the fluidizing effect exhibited in bacterial membrane models. Although 30 ns is a relatively short simulation time, it is sufficient to understand the behavior of the system since a trend is clearly defined from 10 ns. Finally, this study serves as a prototype for better understanding of the interactions between these kinds of molecules and biological membranes, as well as opening prospects for future work in this area. 

## Figures and Tables

**Table 1:** T_m_, ΔT_m_/2, and enthalpy transition (ΔH) values of MLVs constituted by DMPC and DMPC/DMPG (3:1) before and after the addition of *Bz-Im* at different *Bz-Im*–lipid molar ratios.

MLV	Compound–Lipid Molar Ratio	Pretransition Temperature (°C)	T_m_ (°C)	ΔT_m_/2 (°C)	ΔH (J·g^−1^)
DMPC	0:1	12.94	23.02	0.98	1.48
DMPC-Compound	1:50	11.58	22.94	1.09	1.05
	1:25	-	22.60	1.14	0.78
	1:10	-	22.38	1.23	0.76
DMPC/DMPG (3:1)	0:1	12.71	23.02	0.73	1.71
DMPC/DMPG (3:1)-Compound	1:50	12.49	22.98	0.90	1.46
	1:25	-	22.24	1.08	1.38
	1:10	-	19.72	2.34	1.13

## Supplementary Materials

The following are available online at https://www.mdpi.com/article/10.3390/membranes11060449/s1. Section 1: Spectral data. Figure S1: Comparative FT-IR spectra of Bz and *Bz-Im*. Figure S2: NMR-1H spectra of Bz. Figure S3: NMR-1H spectra of *Bz-Im*. Figure S4: NMR-COSY spectra of *Bz-Im*. Figure S5: NMR-13C{1H} spectra of *Bz-Im*. Figure S6: NMR-HSQC spectra of *Bz-Im*. Figure S7: NMR-HMBC spectra of *Bz-Im*. Figure S8: Mass spectra (EI) of Bz. Figure S9: Mass spectra (DART+) of *Bz-Im* [M+1]; Section 2: Figure S10: Root-mean-square deviation (RMSD) of *Bz-Im* in the system. Figure S11: Hydrogen bonds between *Bz-Im* and the phospholipids of the system.
