# Supplementary material for: A Study of the Interaction of a New Benzimidazole Schiff Base with Synthetic and Simulated Membrane Models of Bacterial and Mammalian Membranes
Source: Membranes (Basel). 2021 Jun 16;11(6):449. doi: 10.3390/membranes11060449 (PMC8235182; doi:10.3390/membranes11060449)
Supplement: Supplementary file 1 [file membranes-11-00449-s001.zip › membranes-1226582-supplementary.pdf]

Supporting Materials for:

A Study of the Interaction of a New Benzimidazole Schiff base with Synthetic and Simulated Membrane Models of Bacterial and Mammalian Membranes

Alberto Aragón-Muriel [1], Yamil Liscano [2], David Morales-Morales [3], Dorian Polo-Cerón [1] and Jose Oñate-Garzón [2,]*

[1] Laboratorio de Investigación en Catálisis y Procesos (LICAP), Departamento de Química, Facultad de Ciencias Naturales y Exactas, Universidad del Valle, 760001 Cali, Colombia; alberto.aragon@correounivalle.edu.co (A.A.-M.); dorian.polo@correounivalle.edu.co (D.P.-C.)
[2] Grupo de Investigación en Química y Biotecnología (QUIBIO), Facultad de Ciencias Básicas, Universidad Santiago de Cali, 760031 Cali, Colombia; yamil.liscano00@usc.edu.co (Y.L.); jose.onate00@usc.edu.co (J.O.-G.)
[3] Instituto de Química, Universidad Nacional Autónoma de México, Cd. Universitaria, Circuito Exterior, Coyoacán, 04510 México D.F., México; damor@unam.mx

* Correspondence: jose.onate00@usc.edu.co

1. Spectral data

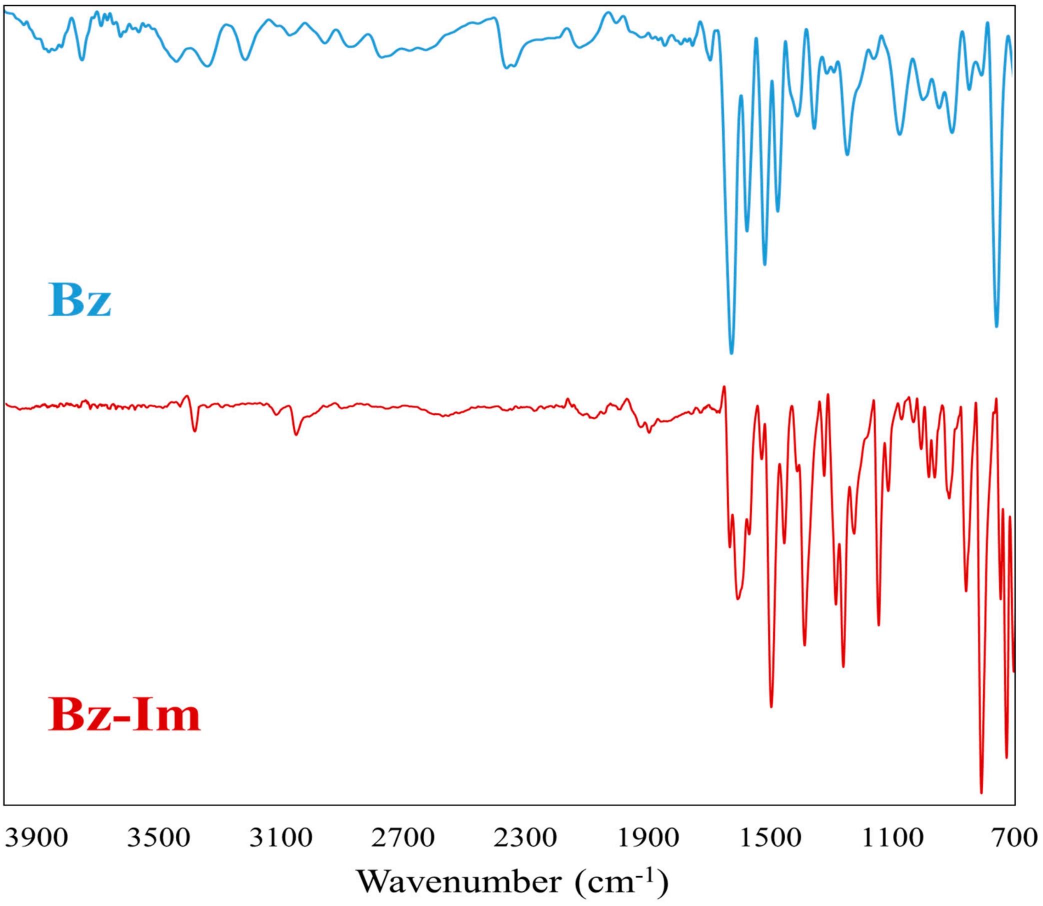

Figure S1. Comparative FT-IR spectra of *Bz* and *Bz-Im*

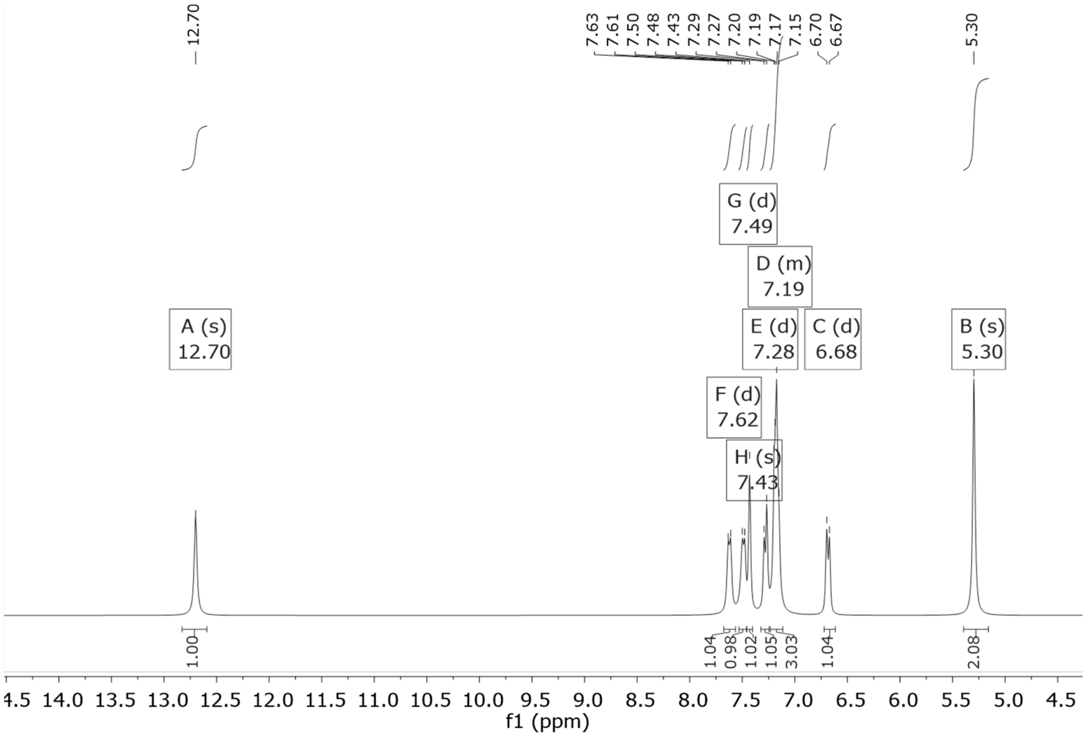

Figure S2. NMR-^{1}H spectra of *Bz*

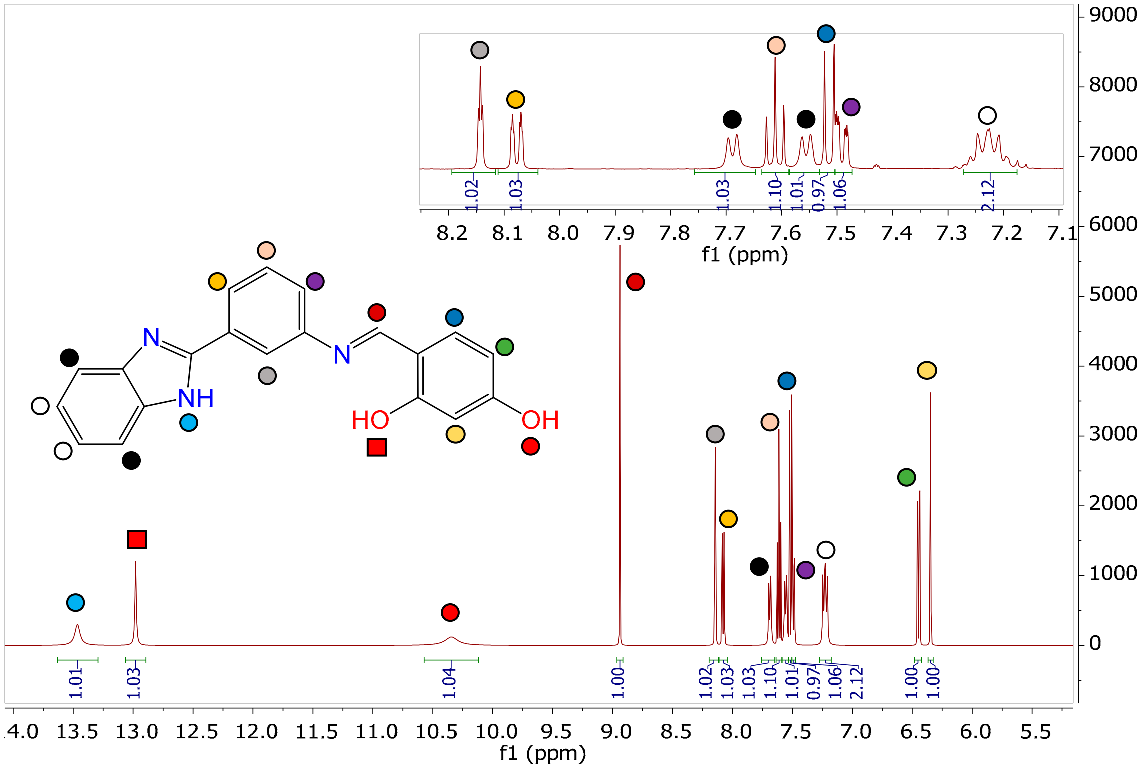

Figure S3. NMR-^{1}H spectra of *Bz-Im*

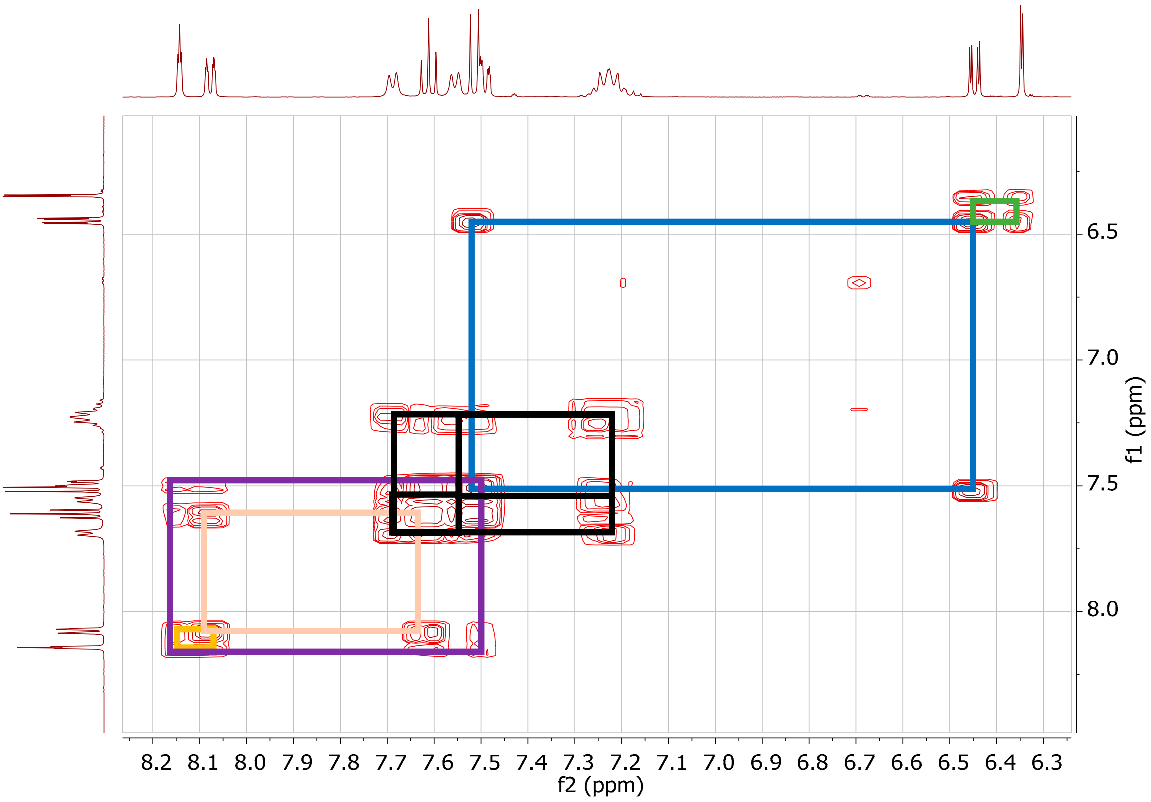

Figure S4. NMR-COSY spectra of *Bz-Im*

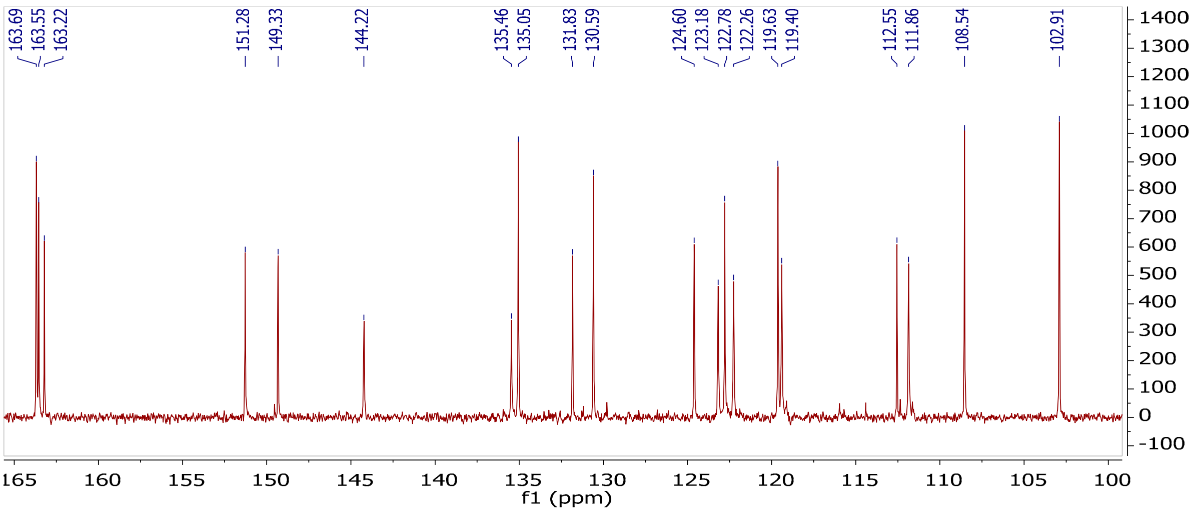

Figure S5. NMR-$^{13}C\{^{1}H\}$ spectra of *Bz-Im*

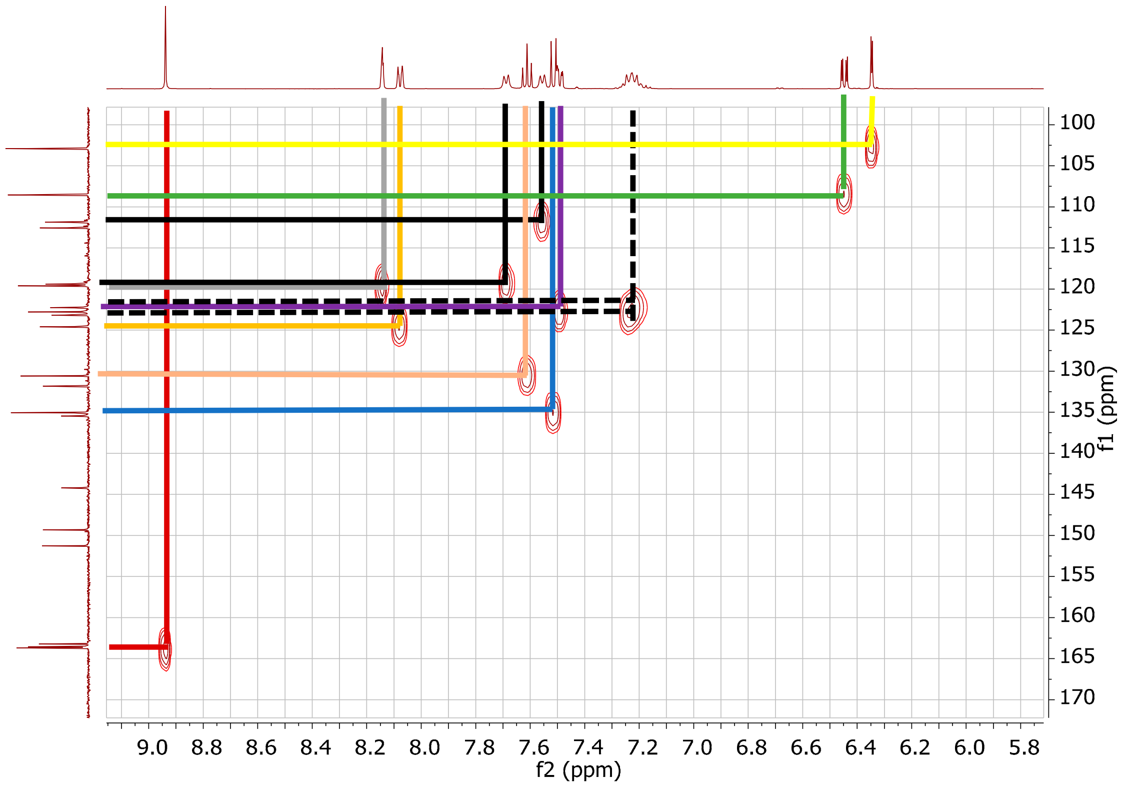

Figure S6. NMR-HSQC spectra of *Bz-Im*

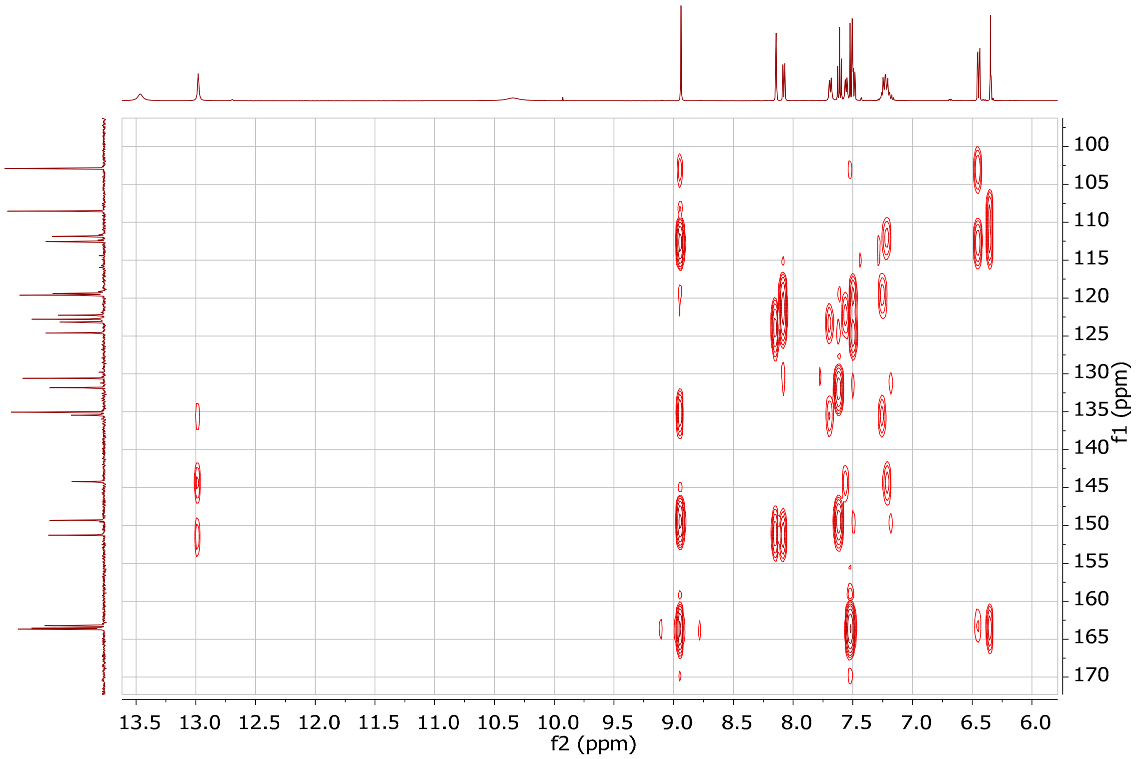

Figure S7. NMR-HMBC spectra of *Bz-Im*

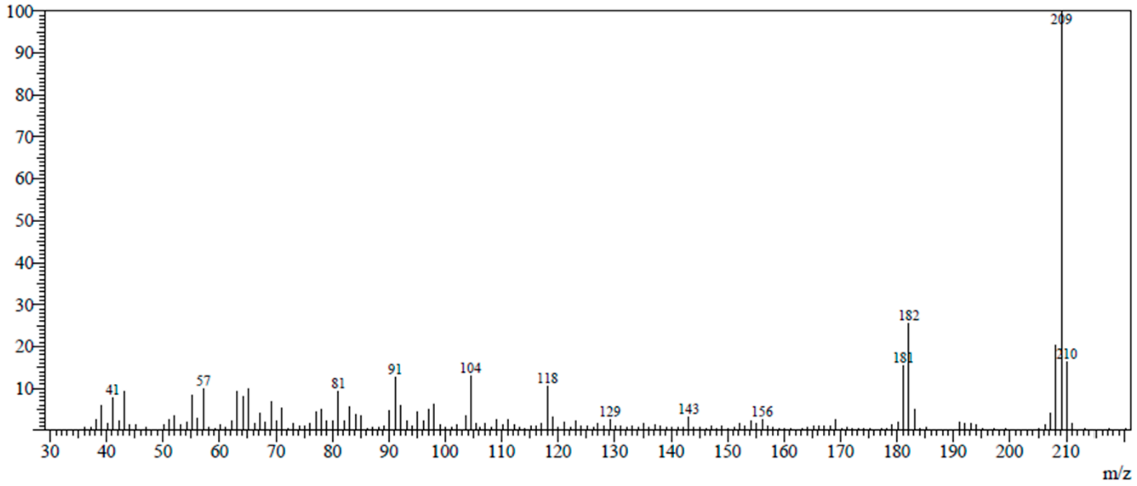

Figure S8. Mass spectra (EI) of *Bz*

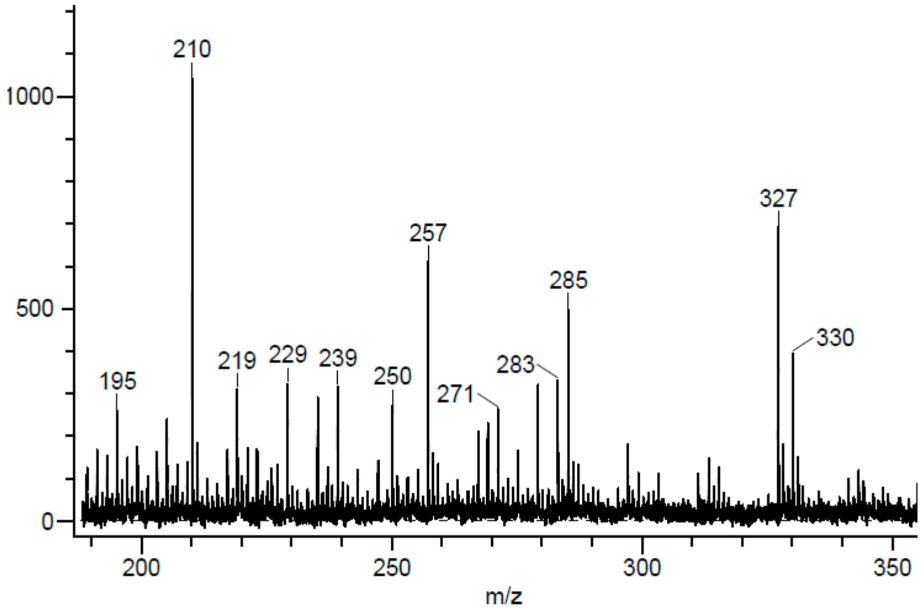

Figure S9. Mass spectra (DART+) of *Bz-Im* [M+1]

2. Molecular Dynamics towards PC-3 cancer cells

2.1. PC-3 Membrane Construction

The model membrane of PC-3 cells was built with the CHARMM-GUI [1] platform using as a basis the phospholipid composition mentioned by Ferreri et al. [2]. The phospholipids used were POPE (1-palmitoyl-2-oleoyl-sn-glycero-3-phosphoethanolamine) with 27 units, POPS (1-palmitoyl-2-oleoyl-sn-glycero-3-phosphoserine) with 10 units and POPC (1-palmitoyl-2-oleoyl-sn-glycero-3-phosphocholine) with 44 units, that were distributed both in the upper and lower layer of the membrane. A Monte Carlo ion placement method with 0.15 M NaCl, with a water thickness of 22.5 Å and as a force field for the entire CHARMM36m system was used [3]. The files were prepared to minimize energy, balance and dynamics with GROMACS [4] at 310 K.

2.2. Implementing Molecular Dynamics

The minimization energy of the PC-3 model membrane and *Bz-Im* system was adjusted with the steepest descent algorithm in 5000 steps and Verlet as *cutoff-scheme*. Equilibration was performed for 2 ns using the Berendsen algorithm to equilibrate the temperature and pressure of the system. Molecular dynamics were run for 10 ns at 310 K using the Nose-Hoover and Parrinello-Rahman algorithms to adjust temperature and pressure. The Monte Carlo method was used with 0.15 M NaCl, a water thickness of 22.5 Å, and CHARMM36m [3] as a force field. The systems were adjusted by slowly heating at 310 K at 1 femtosecond (fs)/step for 75 picoseconds (ps) at 2 fs/step for 300 ps in the equilibration step. Energy minimization of the system was achieved using the steepest descent algorithm with a tolerance value of 1000 kJ mol^{-1} nm^{-1} in 5000 steps with the Verlet *cutoff scheme.* The Berendsen algorithm to 155000 n-steps was used to equilibrate the temperature and pressure of the system. When the system was equilibrated, the production MD for data collection was run for 10 nanoseconds (ns) using the Nose–Hoover and Parrinello–Rahman algorithms to adjust the temperature and pressure. Particle Mesh Ewald (PME) summation was applied to correct for long-range electrostatic interactions.

2.3. Interaction analysis

Gromacs was used to obtain the hydrogen bonds between *Bz-Im* and the phospholipids of the membrane model system within 10 ns. pymol PDB files were obtained for each membrane system at four different times, 1, 4, 8 and 10 ns. These files were used to visualize and analyze the interactions between the different components of each model system and the *Bz-Im* using Discovery Studio Visualizer software (http://accelrys.com).

2.4. Analysis of Molecular Dynamics

In order to identify the selectivity of *Bz-Im* towards PC-3 cancer cells, molecular dynamics were performed on the membrane model using POPE, POPC and POPS, the latter increasing its expression in cancer cells unlike a normal cell. Hence, if *Bz-Im* has a greater interaction with POPS, it might indicate a tendency for an increased cytotoxic effect towards cancer cells. The behavior of *Bz-Im* in the system was first observed for 10 ns with the RMSD. Between 0 ns and 4 ns, the RMSD of *Bz-Im* showed a variation in its structure, as a result of interactions with the surface of phospholipids (Figure S10). At 4 ns, the *Bz-Im* structure is maintained with a slight variation in RMSD,

corresponding to the surface contact and internalization of *Bz-Im* inside the head polar group of phospholipids. Staying in it without reaching the inner phospholipid monolayer or going backout to the aqueous environment.

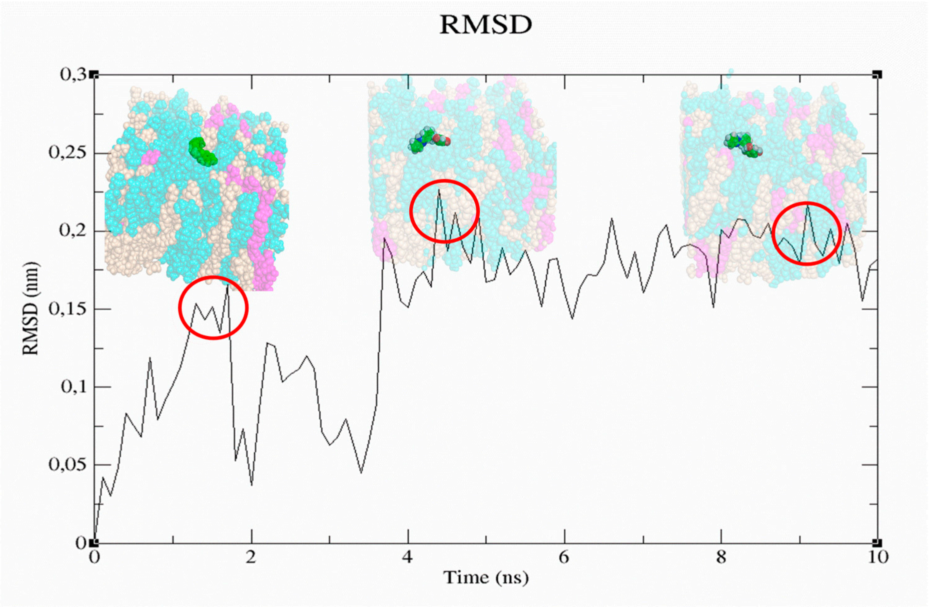

Figure S10. Root Mean Squared Deviation (RMSD) of *Bz-Im* in the system. In green *Bz-Im*, in blue POPC, in violet POPS, and in orange POPE. The systems in red circles represent the position of *Bz-Im* over time.

On figure S11, the number of hydrogen bonds that *Bz-Im* form with phospholipids is illustrated. In the first instance, it can be observed that there are no interactions with POPS. *Bz-Im* forms in the first nanosecond up to three hydrogen bonds with POPE and POPC. This occurs again for POPC at 5 ns and for POPE at 8 ns. However, throughout the 10 nanoseconds there is a trend to form at least two hydrogen bonds with POPC, this being probably due to the 44 units of this phospholipid in the system compared with the 27 of POPE.

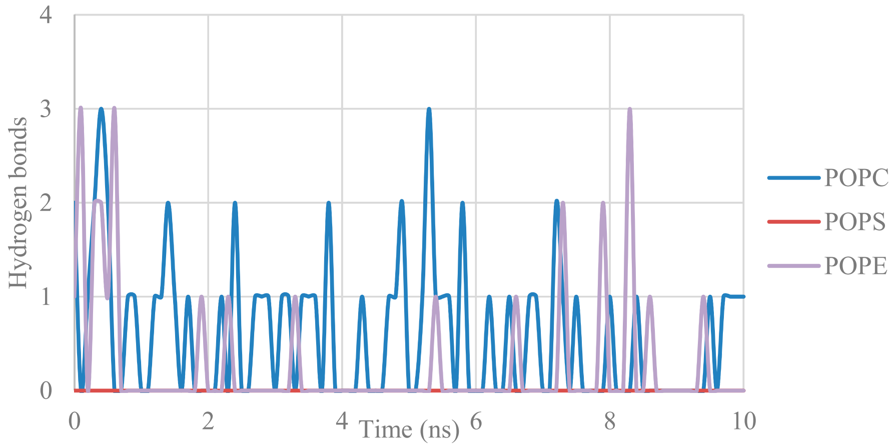

Figure S11. Hydrogen bonds between *Bz-Im* and the phospholipids of the system.

On figure S12, the hydrogen bonds formed between *Bz-Im* and other molecules in the system are shown. At 2 ns there are interactions with water molecules, at 5 ns the same interactions are still observed. At 9 ns, the interaction of the imino-nitrogen atom of *Bz-Im* with the oxygen of the carbonyl of POPE. In general, low affinity is observed by *Bz-Im* for membranes that mimic those of cancer cells on the *in silico* level. In this way, at an experimental level this result could be interpreted as the compound *Bz-Im* to have a similar cytotoxic activity with normal and cancer cells, which, could be related to the effects observed on the phase transition in model membranes imitating mammalian described above.

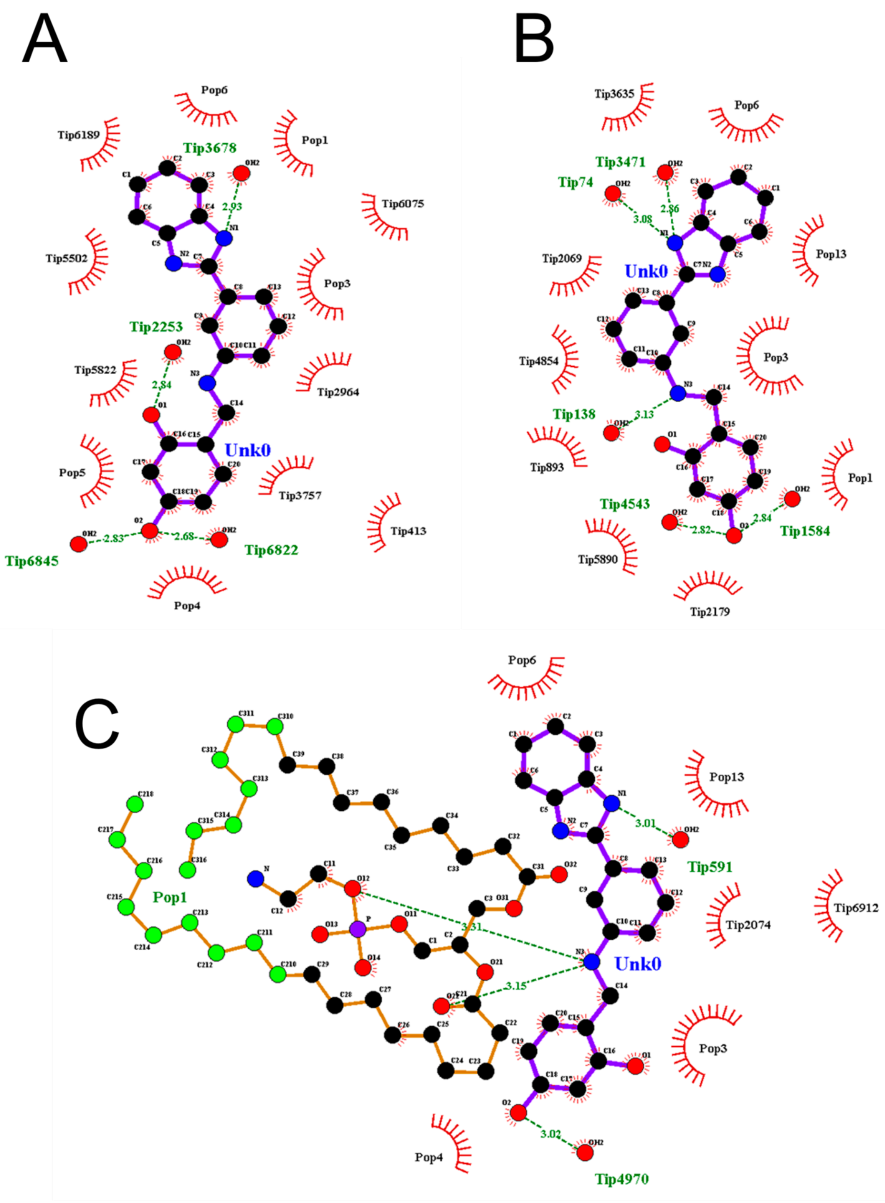

Figure S12. Interaction of *Bz-Im* with the molecules of the cancer membrane model system (A) At 2 ns. (B) At 5 ns. (C) At 9 ns. TIP: water molecule. Unk0: *Bz-lm*. Pop1: POPE.

References

1. Jo, S.; Kim, T.; Iyer, V.G.; Im, W. CHARMM-GUI: A web-based graphical user interface for CHARMM. *J. Comput. Chem.* **2008**, *29*, 1859–1865, doi:10.1002/jcc.20945.

2. Ferreri, C.; Sansone, A.; Buratta, S.; Urbanelli, L.; Costanzi, E.; Emiliani, C.; Chatgilialoglu, C. The n-10 fatty acids family in the lipidome of human prostatic adenocarcinoma cell membranes and extracellular vesicles. *Cancers (Basel).* **2020**, *12*, 1–16, doi:10.3390/cancers12040900.

3. Huang, J.; Mackerell, A.D. CHARMM36 all-atom additive protein force field: Validation based on comparison to NMR data. *J. Comput. Chem.* **2013**, *34*, 2135–2145, doi:10.1002/jcc.23354.

4. Abraham, M.J.; Murtola, T.; Schulz, R.; Páll, S.; Smith, J.C.; Hess, B.; Lindah, E. Gromacs: High performance molecular simulations through multi-level parallelism from laptops to supercomputers. *SoftwareX* **2015**, *1–2*, 19–25, doi:10.1016/j.softx.2015.06.001.
